# Greater Anteroposterior Default Mode Network Functional Connectivity in Long-Term Elderly Yoga Practitioners

**DOI:** 10.3389/fnagi.2019.00158

**Published:** 2019-07-02

**Authors:** Danilo Forghieri Santaella, Joana Bisol Balardin, Rui Ferreira Afonso, Giuliana Martinatti Giorjiani, João Ricardo Sato, Shirley Silva Lacerda, Edson Amaro Jr., Sara Lazar, Elisa H. Kozasa

**Affiliations:** ^1^Hospital Israelita Albert Einstein (HIAE), São Paulo, Brazil; ^2^Centro de Práticas Esportivas da Universidade de São Paulo (CEPEUSP), São Paulo, Brazil; ^3^Center for Mathematics, Computing and Cognition—Universidade Federal do ABC (UFABC), Santo André, Brazil; ^4^Massachusetts General Hospital, Harvard Medical School, Boston, MA, United States

**Keywords:** functional connectivity, yoga, elderly, default mode network, angular gyrus, health aging, cognition

## Abstract

Large-scale brain networks exhibit changes in functional connectivity during the aging process. Recent literature data suggests that Yoga and other contemplative practices may revert, at least in part, some of the aging effects in brain functional connectivity, including the Default Mode Network (DMN). The aim of this cross-sectional investigation was to compare resting-state functional connectivity of the medial prefrontal cortex (MPFC) and posterior cingulate cortex—precuneus (PCC-Precuneus) in long-term elderly Yoga practitioners and healthy paired Yoga-naïve controls. Two paired groups: yoga (Y-20 women, Hatha Yoga practitioners; practicing a minimum of twice a week with a frequency of at least 8 years) and a control group (C-20 women, Yoga-naïve, matched by age, years of formal education, and physical activity) were evaluated for: Mini Mental State Examination (MMSE), Beck Depression Inventory (BDI), Instrumental Activities of Daily Living (IADL), and open-eyes resting-state functional magnetic resonance imaging (fMRI)—seed to voxel connectivity analysis (CONN toolbox 17.f) with pre-processing—realignment and unwarping, slice-timing correction, segmentation, normalization, outlier detection, and spatial filtering. The analysis included *a priori* regions of interest (ROI) of DMN main nodes—MPFC and PCC-Precuneus. There was no difference between groups in terms of: age, years of formal education, MMSE, BDI and IADL. The Yoga group had a higher correlation between MPFC and the right angular gyrus (AGr), compared to the controls. Elderly women with at least 8 years of yoga practice presented greater intra-network anteroposterior brain functional connectivity of the DMN. This finding may contribute to the understanding of the influences of practicing Yoga for a healthier cognitive aging process.

## Introduction

Life expectancy constantly increases worldwide. The elderly population will increase from 11% to 22% of the world’s total population by 2050 (WHO, 2014). During the aging process, the human brain suffers functional connectivity losses, which may include decreased connectivity of the default mode (Mowinckel et al., 2012), working memory (DeCarli et al., 2012), and salience (He et al., 2014) networks, as well as the decreased resting-state connectivity of the hippocampus (Salami et al., 2014). Further evidence of age-associated cognitive decline has been provided in studies which show the decreased functional connectivity between the anterior and posterior structures of DMN (default mode network; Toussaint et al., 2014; Vidal-Piñeiro et al., 2014) as well as smaller deactivations of the same network when facing attentional stimuli (the inferior and superior frontal gyrus, posterior cingulate gyrus, medium temporal gyrus and the superior parietal cortex; Damoiseaux et al., 2008). Another study showed a reduced inter-network connectivity among salience network (SN), DMN, and the central executive network in elderly volunteers—weakened connectivity was found between the frontal insular cortex and the anterior cingulate cortex (He et al., 2014). Such connectivity decreases account for the progressive cognitive loss of the aging process (Hafkemeijer et al., 2012), reflecting the need for research efforts to find effective low-cost and low side-effect methods to counteract them.

Yoga has been widely studied among contemplative practices and may be cognitively beneficial for the elderly. It differs from other types of western physical exercises as great importance is given to attentional processes. Yoga practice is based on postures (asanas) and breathing exercises (pranayamas), which must be performed with a great attentional component and without tension, leading to concentration (dharana) and meditation (dhyana); additionally, great importance is given to attitudinal prescriptions (yamas and niyamas), such as non-violence, truth and contentment; practical components which must be applied during postures and breathing exercises (Taimni, 1961).

Although Yoga is different than Western exercises (it cannot be considered just an Indian approach towards physical exercise, since it is contemplative in its very nature), its physical and psychological effects seem to be equal or, in some cases, even greater than many psychophysiological health indicators such as aerobic exercises (Ross and Thomas, 2010). In fact, some authors have verified an increased fluid intelligence and greater functional resilience in elderly who practice Yoga or meditation (Gard et al., 2014b). In another study (Gard et al., 2015), the authors found greater widespread resting-state functional connectivity (increased degree centrality) of the caudate in an elderly sample who practiced yoga and meditation, compared to a control group; such increases were present mainly between the parahippocampal gyrus and the left inferior temporal gyrus, suggesting effects of these practices in motor processes, procedural and associative learning, and the inhibitory control of the action and reward system. Furthermore, meditators have a more robust connection between the posterior cingulate and dorsolateral prefrontal cortex (DPFC), which may indicate cognitive preservation (Brewer et al., 2011). Nevertheless, there is still a lack of evidence on the long-term effects of Yoga practice on functional resting-state connectivity. One study, which included mildly cognitive impaired patients, showed that 12 weeks of Yoga practice was as effective as memory enhancement training, positively influencing resting-state functional connectivity between the DMN and some brain areas related to verbal memory performance; the main areas included the frontal medial cortex, posterior cingulate cortex (PCC), pregenual anterior cingulate cortex and the right middle frontal cortex (Eyre et al., 2016). However, the influence of longer periods of attentional practice, such as in Yoga, on resting-state functional connectivity has not been investigated yet. Structural imaging investigations have shown the positive long-term effects of Yoga. Some authors have found increased gray matter associated to long-term Sahaja Yoga (Hernández et al., 2016), results which strengthen those of our group, showing an increased cortical thickness of the prefrontal cortex (PFC) in long-term elderly Yoga practitioners (Afonso et al., 2017). Altogether, these characteristics and findings suggest that Yoga is a very comprehensive attentional training method and may increase functional connectivity of the DMN of the elderly population. There is a consensus in the literature concerning the existence of an anterior and a posterior part of the DMN, each of them having a main area which may be termed as its main hub. The medial prefrontal cortex (MPFC) is the anterior hub, and the posterior cingulate cortex—precuneus (PCC-Precuneus), the posterior hub (Whitfield-Gabrieli and Ford, 2012). Following this observation, we aimed at investigating MPFC and PCC-Precuneus as the two seed-based regions of interest (ROIs) for functional connectivity analysis. We hypothesize that long-term elderly Yoga practitioners with at least 8 years of regular practice have increased anteroposterior resting-state functional connectivity compared to paired Yoga naïve, non-practitioners.

## Materials and Methods

### Participants

The study protocol was approved by the Institutional Review Board of Hospital Israelita Albert Einstein (CAAE 64633317.7.0000.0071). All volunteers provided written informed consent to participate in the protocol. In order to exclude the influence of physical fitness and years of formal education on functional connectivity, this study aimed at paired groups. Participants of the Yoga group (Y), were recruited from Hatha Yoga studios in São Paulo, Brazil. Yoga volunteers were included if they practiced Yoga at a minimum frequency of twice a week for at least 8 years. The Yoga group consisted of 20 women, which were paired to a control group (C), formed by an additional 20 women, naïve to Yoga, meditation or any mind-body intervention. Controls were matched to the first group by age, years of formal education, type and level of physical activity. Those in the Yoga group who practiced only Yoga and no other physical activities were paired with sedentary volunteers in the control group; subjects who practiced Yoga along with other physical activities were paired with the Yoga naïve subjects, with the same type of physical activity in the control group. Inclusion criteria: age equal or greater than 60 years; right-handed female; at least elementary school completed. The criterion of including only women was chosen in order to assure a greater homogeneity to the group. Exclusion criteria: substance abuse; tremor or dystonia of the head; chronic physical problems or other health issues that might prevent volunteers from performing daily activities independently; any contraindication to magnetic resonance imaging (MRI); clinical history of neurological and/or psychiatric diseases, claustrophobia, and uncontrolled health problems (diabetes, cardiopathy, neoplasia). All participants underwent the Mini Mental State Examination (MMSE), which is a widely applied and validated test to address cognitive dysfunction (Nilsson, 2007), and has sensitivity to exclude dementia. In order to ensure a better psychological matching between groups, and also to avoid psychological illness, Beck Depression Inventory (BDI) was also applied.

### Questionnaires and Tests

#### Mini Mental State Examination (MMSE)

This test evaluates cognitive function in domains such as spatial-temporal orientation; language-naming; calculation; writing; repetition and copying; immediate and evoked memory. Its application results in scores which range from 0 to 30 (Folstein et al., 1975).

#### Beck Depression Inventory (BDI)

Self-reported questionnaire, which has 21 multiple choice questions (scored from 0 to 3) to address depression symptoms. Total scores range from 0 to 63 (Beck, 1978; Gorenstein et al., 1999).

#### Instrumental Activities of Daily Living (IADL)

The ability of the participant in performing each task (independently; with the help of others or not at all) is scaled and presented as results in a score at each item evaluated. Scores close to 9 are considered as indicative of a low function, while a high function is given by scores closer to 27 (Lawton and Brody, 1969; Santos and Virtuoso, 2008).

Weight and height were measured as anthropometric variables.

### Image Acquisition

MRI data were collected on a 3.0T MR system (Siemens Tim Trio, Erlanger, Germany) using a 12-channel head coil. Subjects were instructed to avoid head movements and to keep their eyes open staring at a fixation cross presented at the center of the visual field. Functional images were acquired in a 7 min scan using a blood oxygen level-dependent (BOLD) sensitive gradient echo-planar-image (GRE-EPI) pulse sequence with the following parameters: 2D oblique axial plane (AC-PC oriented); 33 slices with 2.4 mm thickness and 0.4 mm gap, in-plane resolution = 3.4 mm × 3.4 mm and flip angle = 90°, whole-brain 150 volumes. High spatial resolution anatomical images were acquired using a T1-weighted MPRAGE sequence with the following parameters: 3D sagittal plane acquisition, TR: 2,500 ms; TE: 3.45 ms; 1 mm isotropic voxels; Flip angle: 7°; acquisition matrix: 256 × 256; NEX: 1.

#### fMRI Analysis

Data were preprocessed and analyzed using the CONN toolbox version 17.f (Whitfield-Gabrieli and Nieto-Castanon, 2012), with a standard MNI152 pipeline and parameters. Preprocessing steps included the realignment and unwarping, slice-timing correction, segmentation, normalization, outlier detection, and smoothing. Nuisance variables were based on scan motion censuring (discarding volumes with displacement >2 mm and global-signal *z*-value >9; no subjects were excluded), realignment parameters (12), white-matter and CSF signals. Band-pass filtering (0.008–0.09 Hz) and nuisance variables were regressed out using a simultaneous bandpass approach (Hallquist et al., 2013).

**Figure 1:**
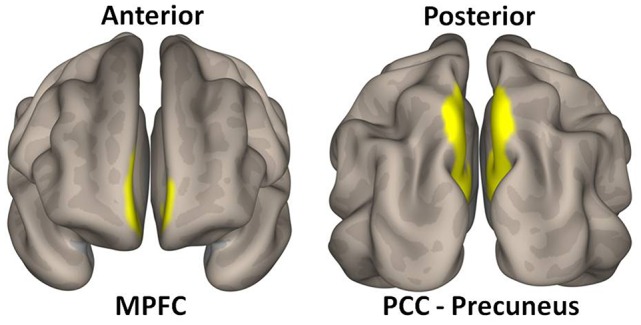
Medial prefrontal cortex (MPFC—MNI coordinates: +1 + 55 −3) and posterior cingulate cortex-precuneus (PCC-precuneus—MNI coordinates: +1 −61 + 38) used as regions of interest (ROIs) for respectively anterior and posterior components of the default mode network (DMN).

DMN main nodes (MPFC and posterior cingulate gyrus—PCC-Precuneus) were *a priori* ROI used as seeds in the resting-state functional connectivity analysis (Figure 1). Seeds were selected from prior studies (Whitfield-Gabrieli and Ford, 2012), and defined with masks from the Harvard-Oxford Atlas^1^.

Connectivity first-level correlation maps were produced by extracting the mean BOLD time course from voxels within each seed and computing Pearson’s correlation coefficients between that time course and the time course of all other voxels. Correlation coefficients were converted to normally distributed *Z*-scores using the Fisher transformation in order to allow second-level General Linear Model analysis. Two sample *t*-tests were performed on the Fisher transformed r-maps to examine differences in resting-state functional connectivity between the Yoga and Control groups. Group-level effects were considered significant if they exceeded a peak amplitude of *t* > 3.09, and a family wise error corrected cluster extent threshold of *p* < 0.05.

## Results

Sample characteristics of the volunteers in the Yoga and Control groups are shown in Table 1—no difference was found between the groups: all volunteers were over 60 years old; with their body mass index (BMI) inside normality range and average years of formal education above the Brazilian average (7.8 years); Mini Mental scores did not indicate any cognitive impairment; BDI scores ranked at minimum, and IADL results were also within normality.

**Table 1:** Sociodemographic characteristics of the volunteers in the Yoga and Control groups.

Characteristics	Control group (*n* = 20)	Yoga group (*n* = 20)	*p*-value
Age (years)	68.2 (4.6)	66.5 (4.5)	0.24
BMI (kg/m^2^)	25.3 (3.0)	24.8 (4.3)	0.66
Education (years)	14.6 (2.0)	14.3 (1.9)	0.63
MMSE	28.8 (1.3)	28.2 (1.8)	0.23
BDI	7.9 (5.7)	5.3 (4.6)	0.13
IADL	26.7 (0.7)	27.0 (0.2)	0.12
Years of yoga practice		15.1 (8.3)	

*Note: data expressed as means (standard deviation); BMI, body mass index; MMSE, Mini-Mental State Examination; BDI, Beck Depression Inventory; IADL, Instrumental Activities of Daily Living; *p*-values obtained by unpaired Student’s *t*-test between groups*.

A graphic representation of DMN ROIs used as seeds for the connectivity analysis is presented in Figure 1.

### Medial Prefrontal Cortex (MPFC)

**Figure 2:**
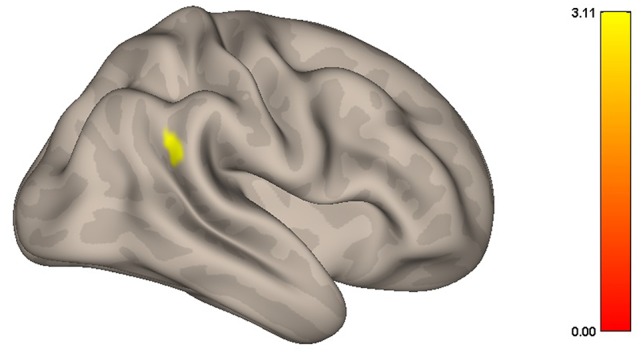
Graphic representation of the resting state functional connectivity of the DMN during resting-state. Women in the Yoga group had significantly greater correlation (*p* < 0.05) between the MPFC and right angular gyrus (AGr) than the Control group.

**Figure 3:**
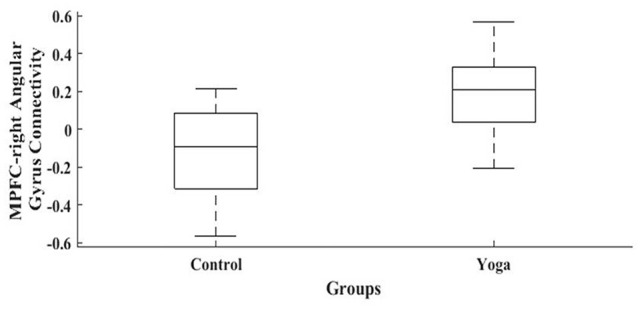
Resting state functional connectivity of the DMN during rest. Women in the Yoga group had significantly greater correlation (*p* < 0.05) between the MPFC and right angular gyrus than the Control group.

When MPFC was used as ROI for functional connectivity analysis, with the contrast Yoga > Control, the Yoga group presented a significantly increased correlation between MPFC and the right angular gyrus (AGr), compared to the Control group. Specific data is as follows: peak-cluster coordinates: +64 −50 + 26; cluster size: 127; cluster *p*-value FWE: 0.042347 (Figures 2, 3). When the contrast Control > Yoga was used, no significant difference was found between the two groups.

### Posterior Cingulate Gyrus—Precuneus (PCC-Precuneus)

There was no significant difference between the two groups when PCC-Precuneus was used as ROI for functional connectivity analysis neither using the contrast Yoga > Control nor Control > Yoga.

## Discussion

In this cross-sectional study, we found greater resting-state anteroposterior functional brain connectivity between the MPFC and the angular gyrus (AGr) in healthy elderly women who practiced Yoga for at least 8 years, when compared to the paired Yoga-naïve controls.

More than mapping specificities of the brain areas and factors which led to their activation, neuroscience evolved to the investigation of relationships of the brain’s inner activity and correlations of actions among its many areas even during resting-state, which allowed more meticulous access to cognitive processes and mechanisms. In fact, our laboratory’s previous work on structural brain magnetic resonance has shown the increased thickness of the right middle frontal gyrus in elderly females with at least 8 years of Hatha Yoga practice (Afonso et al., 2017), strengthening the findings of some recent studies (Brewer et al., 2011; Gard et al., 2015; Eyre et al., 2016) which indicate the influence of attentional practices such as meditation and Yoga on preserving cognition and brain functional connectivity. Those findings motivated us to address the possibility of a resting-state functional connectivity difference between Yoga practitioners and controls.

Many authors have shown a decrease in brain resting-state functional connectivity in the elderly (Damoiseaux et al., 2008; DeCarli et al., 2012; Mowinckel et al., 2012; He et al., 2014; Toussaint et al., 2014; Vidal-Piñeiro et al., 2014), which may account for the reduction of cognitive flexibility presented during the senescence process. More specifically, when the DMN comes into focus, some authors state that senescence leads to a decreased anteroposterior connectivity (Toussaint et al., 2014; Vidal-Piñeiro et al., 2014). Our results show an intrinsic preservation of functional brain connectivity between MPFC and AGr during resting-state in the group composed of elderly women who practiced yoga for at least 8 years. In fact, some authors have demonstrated the positive effects of contemplative practices in preserving functional brain resting-state connectivity. Brewer et al. (2011) have reported increased functional resting-state connectivity between mPCC and the fusiform gyrus, inferior temporal gyrus, inferior parietal lobe and the cerebellum in adults who practiced at least 10 years of meditation; differences in results may be due to between-sample differences, such as a greater amount of hours of practice between their volunteers and ours; differences in age or differences between meditation types investigated and Yoga—nevertheless, results point to a positive relationship between meditation and yoga on DMN connectivity. Additionally, Gard et al. (2015) have shown that both elderly yoga practitioners and meditators have greater widespread functional connectivity of the caudate, compared to matched controls. The same authors have also found that those elderly who practice yoga or meditation, have resting-state functional networks that are more integrated and more resilient to damage than the control subjects, indicating a greater resilience and also a decreased speed of fluid intelligence decline during the senescence process (Gard et al., 2014b).

Furthermore, the specific structures which presented greater connectivity in our study were the MPFC and the angular gyrus, which comprise the anterior and posterior nodes of the DMN, respectively. The angular gyrus is well known to be associated with reading and associative memory, in addition to its important role in directing attention, particularly in bottom-up processes (Studer et al., 2014), which are trained during Yoga practice (Gard et al., 2014a). Additionally, it is considered to be responsible for action-outcome monitoring, related to action control and feedback, being a supramodal area, and also functionally connected with self-referential areas such as the PCC and precuneus during delay detection (visual, auditory and audio-visual; van Kemenade et al., 2017), processes which are continuously entrained and influenced by Yoga practice (Gard et al., 2014a; Acevedo et al., 2016) These findings suggest that Yoga may have a protective effect on cognition (Afonso et al., 2017; Gothe et al., 2018).

It is important to stress the fact that all yoga practices under the classification “asanas” (postures) and “pranayamas” (breathing exercises), which are the main components of routines practiced along years of training in Yoga classes, are attentional in their very nature, and serve as a preparation for the meditative practices which invariably follow them—achieving a mindful or meditative state of mind is the main objective of the Yoga system, which is based on self-discipline (Taimni, 1961). As such, it is not a surprise to find increased connectivity between regions of the DMN, which is more active during rest and self-referenced planning. Additionally, the angular gyrus is physically connected to the prefrontal region through the superior longitudinal fasciculus, and also to the caudate (part of the motor network) through the occipitofrontal fasciculus, which may bring up the possibility of a cause-consequence relationship between attention to body parts during the Yoga practice and the increased connectivity of the Yoga group. Such a possibility is also corroborated by the results of another study (Hernández et al., 2018), which found greater functional connectivity between the anterior cingulate cortex and bilateral anterior insula/putamen during a meditation-state.

To the best of our knowledge, the increased resting-state connectivity between anteroposterior areas of the DMN in long-term healthy elderly Yoga practitioners has not been demonstrated in the literature to date, and may form one of the important contributions of this work to the scope of resting-state functional connectivity influences of Yoga practice in the elderly. Such findings are consistent with the hypothesis that training focused attention on comfortable sensations perceived during stretching and body-related respiratory movements during the permanence in postures of Hatha Yoga, named asanas may entrain brain pathways and/or systems which otherwise would gradually become less and less active along the aging process, helping to preserve brain connectivity and self-consciousness, contributing to being healthier during old-age.

This work, however, has its limitations: our sample could have been bigger, which would have strengthened its statistical power. Additionally, since it is a cross-sectional study, and not a longitudinal one, it is not possible to infer a cause-consequence relationship between years of Yoga practice and resting-state functional connectivity of the DMN. Those from the Yoga group who practiced no other type of physical activity were matched to sedentary controls; the small amount of energy expenditure present during Yoga practice has probably not lead to some sort of matching bias, since there was no motor cortex difference between Yoga practitioners and matched controls in a previous structural MR study of our laboratory, which adds to the validity of our matching procedure (Afonso et al., 2017).

In conclusion, elderly women with at least 8 years of yoga practice present greater intra-network anteroposterior brain resting-state functional connectivity of the DMN. This finding may contribute to a better understanding of the influences of Yoga practice for a healthier cognitive aging process.

## Data Availability

The raw data supporting the conclusions of this manuscript will be made available by the authors, without undue reservation, to any qualified researcher.

## Ethics Statement

The study protocol was approved by the Institutional Review Board of Hospital Israelita Albert Einstein (CAAE 64633317.7.0000.0071).

## Author Contributions

DS: acquisition of data, design, interpretation, revising and final approval of the article and agreement to be accountable for all aspects of the work. JB and JS: analysis, revising and final approval of the article and agreement to be accountable for all aspects of the work. RA: acquisition of data, revising and final approval of the article and agreement to be accountable for all aspects of the work. GG, EA, SSL and SL: revising and final approval of the article and agreement to be accountable for all aspects of the work. EK: conception and design of the work, analysis and interpretation of data, revising and final approval of the article and agreement to be accountable for all aspects of the work.

## Conflict of Interest Statement

The authors declare that the research was conducted in the absence of any commercial or financial relationships that could be construed as a potential conflict of interest.

## Footnotes

^1^http://neuro.debian.net/pkgs/fsl-harvard-oxford-atlases.html
